# Opacity in ground glass: remember the gravity-dependent atelectasis

**DOI:** 10.1590/1806-9282.20251426

**Published:** 2025-12-08

**Authors:** Claudio Marcio Amaral de Oliveira Lima, Antônio Carlos Coutinho, Edson Marchiori

**Affiliations:** 1 Hospital Municipal Ronaldo Gazolla – Rio de Janeiro (RJ), Brazil.; 2 Clínica de Diagnóstico por Imagem – Rio de Janeiro (RJ), Brazil.; 3 Universidade Federal do Rio de Janeiro, Department of Radiology – Rio de Janeiro (RJ), Brazil.

Dear Editor,

We read with great interest the study by Shu et al.^1^, which demonstrated that a multi-criteria decision analysis (MCDA), guided bundled intervention, supported by early chest computed tomography (CT) detection, is highly effective in reducing stroke-associated pneumonia (SAP) incidence in acute ischemic stroke patients.

The article presents chest CT images highlighting areas of ground-glass opacities (GGOs) in the lower lobes and concludes that they are indicative of the initial signs of SAP. As radiologists, we disagree with the interpretation of these opacities and would like to make some comments.

GGO is a descriptive term referring to an area of increased attenuation in the lung on CT with preserved bronchial and vascular markings. This finding is nonspecific and has a wide etiology, including infection, chronic interstitial disease, and acute alveolar disease^2–4^. In specific circumstances, GGO may be a manifestation of gravity-dependent atelectasis, resulting from a synergy of diminished alveolar volume and augmented perfusion in the dependent lung regions. This process culminates in the collapse of air sacs, manifesting as a dependent and subpleural distribution. This presentation is frequently observed in the posterior lung bases on CT scans, especially in elderly individuals afflicted with pneumonia or pulmonary edema^3–5^.

It is suggested by the experience of the present authors that the tomographic finding presented must be primarily related to gravity-dependent atelectasis, especially in patients without marked evidence of an infectious process.

## Data Availability

The datasets generated and/or analyzed during the current study are available from the corresponding author upon reasonable request.
